# Non-invasive measurement of rat auditory evoked fields using an optically pumped atomic magnetometer: Effects of task manipulation

**DOI:** 10.1016/j.heliyon.2024.e31740

**Published:** 2024-05-23

**Authors:** Yi Ruan, Zhao Xiang, Guanzhong Lu, Yuhai Chen, Yufei Liu, Fan Liu, Jiahao Wang, Ying Zhang, Jia Yao, Yu Liu, Qiang Lin

**Affiliations:** a Zhejiang Provincial Key Laboratory and Collaborative Innovation Center for Quantum Precision Measurement, College of Science, Zhejiang University of Technology, Hangzhou, 310023, China; b Department of Breast Surgery, The First Affiliated Hospital of Zhejiang University School of Medicine, Hangzhou, 311121, China; c Department of Endocrinology and Metabolism, Shaoxing Second Hospital, Shaoxing, 312000, China

**Keywords:** Magnetoencephalography, Attention, Atomic magnetometer, Passive paradigm, Frequency

## Abstract

Optically pumped magnetometers (OPMs) have become a favorable tool for magnetoencephalography (MEG) measurement, offering a non-invasive method of measurement. OPMs do not require cryogenic environments, sensors can be more closely aligned with the brain. We employed a passive single-stimulus paradigm in conjunction with OPMs with a sensitivity of 20 fT/ $\sqrt{\text{Hz}}$ to investigate the auditory response of rats to inter-stimulus interval (ISI) and frequencies, recording the rat auditory event-related magnetic fields (ERMFs). Our findings include: (1) Auditory evoked fields can be detected non-invasively by OPMs; (2) The amplitude of the rat auditory ERMFs varies with changes in ISI, with more pronounced amplitude changes observed after 5 s; (3) When the sound stimulus frequency is altered at the same ISI, the amplitude of the rats ERMFs changes with frequency, indicating significant differences in attention. Our method offers a valuable tool for the clinical application of a single stimulus paradigm and opens up a new avenue for research on the brain magnetic field detections.

## 1 Introduction

Event-related potentials (ERPs) are average brain electrical potentials triggered by sensory stimuli and time-locked, used to investigate information processing, such as auditory stimuli like pure tones, clicks, or speech, leading to component deflections determined by polarity and occurrence sequence. In traditional ERPs research, classical paradigms are typically employed to elicit responses. A N1 negative wave appears 85–170 ms after the stimulus onset, a positive wave known as P2 emerges between 170 and 260 ms, and potentials appearing after 300 ms are identified as P3. N1 and P2 are believed to exhibit distinct sensitivities to stimulus characteristics such as ISI, sound frequency, and loudness, thereby reflecting involvement in attentional processes [[1], [2], [3], [4]]. P3 is considered a cognitive event associated with tasks necessary for maintaining working memory [5], as this potential is thought to reflect the processes involved in updating the mental model of the stimulus environment [6]. A positive correlation between P3 latency and the degree of cognitive decline has been observed in various clinical populations. Integrating these findings, they suggest that the P3 latency is related to the timing of attention allocation processes, which vary among different individuals [[7], [8], [9]]. The ISI is defined as the time duration between multiple consecutive stimuli, starting from the onset of the first stimulus and ending at the onset of the next stimulus [10]. Investigations in humans have explored ERPs component amplitudes under long and short ISI [[11], [12], [13]], indicating a need for at least 6–10 s of ISI to elicit maximal N1 and P2 amplitudes [14,15].

Traditional studies employ active response paradigms, demanding high participant involvement, requiring attention to each target stimulus and appropriate responses [16,17]. In the case of rodent models, extensive training time is needed. To simplify the paradigm and relieve participants from actively responding to target stimuli, ones have introduced a paradigm involving passive single-stimulus presentation, where target tones are randomly presented, akin to oddball paradigms, but standard tones are replaced by silence. Participants are instructed to respond to target stimuli. Compared to oddball paradigms, the single-stimulus task is simpler, as participants only need to respond to target stimuli. Since target stimuli are infrequent and participants respond in the same manner as oddball tasks, this “single-stimulus” paradigm essentially lacks standard tone oddity. The single-stimulus task and response demands are less complex than oddball paradigms, offering several advantages: 1) The task context is straightforward, requiring responses only upon stimulus occurrence; 2) Participants need to focus attention solely on target stimuli, generating ERPs in a manner similar to oddball paradigms, compared to active task environments; 3) Obtaining and averaging ERPs for individual stimuli simplify electrophysiological recording methods. These factors indicate that passive single-stimulus paradigms can yield reliable ERPs, playing a crucial role in assessing infants and cognitively impaired patients [[18], [19], [20]]. However, in the context of rodent models, there is limited research in this area. In depth studies on rodents are essential, as this research is crucial in forming the foundation for the neurophysiological cognitive processes.

Over the past decade, technological advancements have significantly enhanced biomagnetic field measurement techniques, with electroencephalography (EEG) and MEG finding applications in clinical research [21,22]. However, EEG is limited by spatial resolution and is susceptible to signal distortion due to the skull's conductivity. In contrast, MEG, by measuring the magnetic fields produced by neural currents on the scalp, explores biological phenomena with excellent signal penetration [23,24], is completely non-invasive, and can accurately locate signal sources within the brain cortex [25]. Traditional brain magnetism measurements primarily rely on Superconducting Quantum Interference Devices (SQUIDs), which have numerous limitations in biomagnetic measurements. For example, SQUIDs require low-temperature conditions to operate, have distance limitations from the brain cortex during measurement, and involve high installation and maintenance costs. Their fixed sensor shape cannot be modified according to brain size, impacting the measurement accuracy [26,27]. Recently, OPMs based on optical and quantum principles have become feasible for biomagnetic field measurements. The Spin-Exchange Relaxation-Free (SERF) magnetometer, based on the atomic spin SERF state effect and employing optical detection of magnetic resonance, has emerged as a mainstream sensor for detecting weak magnetic fields such as biomagnetic fields, brain magnetism and cardiac magnetism. In subsequent research, the Romalis group introduced an elliptically polarized light-based SERF magnetometer with a sensitivity of 7 fT/ $\sqrt{\text{Hz}}$, using a compact 5 mm × 5 mm × 5 mm Rb vapor cell, making the device more portable and suitable for confined spaces [28]. Further developments by the SERF Atomic Magnetometer (AM) experimental group at Princeton University involved heating the K vapor cell to 200 °C, achieving a sensitivity of 0.16 fT/ $\sqrt{\text{Hz}}$ [29]. With a volume of only 0.45 cm^3^, much smaller than SQUIDs magnetometers, each sensor can be arrayed according to different brain shapes for a closer fit to the human head, facilitating wearable implementation [[30], [31], [32]].

The objective of this study is to integrate a self-made single-light SERF AM with the neurophysiological signal responses of rats. We evaluated the feasibility of rats under a passive single-stimulus paradigm and explored the impact of ISI and frequencies on the amplitude of related components within this paradigm. This approach provides a new method for subsequent research into human cognitive processes and also further bridges the gap in cognitive information between rats and humans.

## 2 SERF AM

**Fig. 1:**
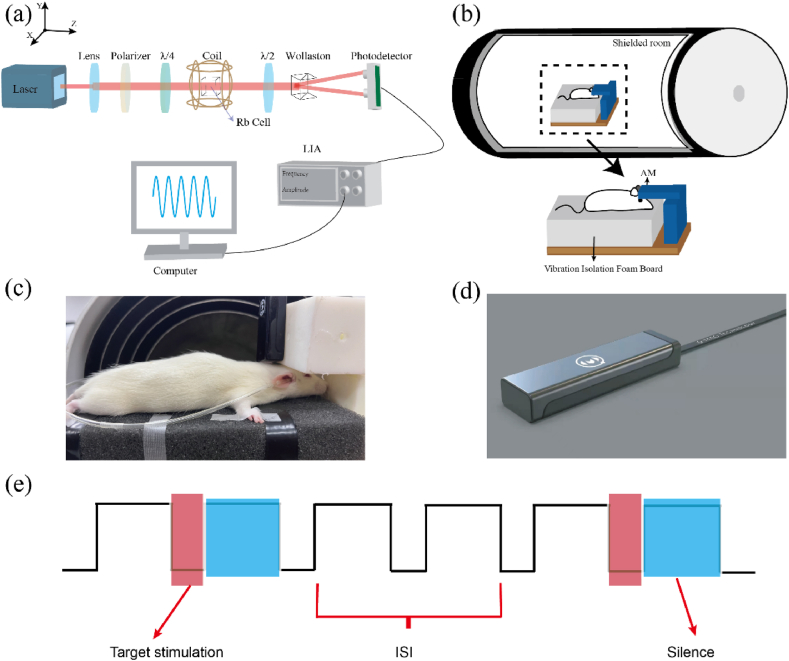
Configuration of the setup. (a) Internal structure diagram of SERF magnetometer. (b) Schematic diagram of rat measurement. A five-layer magnetic shielding was used, incorporating a platform made of silica aerogel pads and wooden boards, both of which are constructed from non-magnetic materials. During the measurement process, the SERF magnetometer and the anesthetized rat were placed inside the shield. (c) Experimental apparatus diagram for rat brain magnetic measurement. The SERF magnetometer is positioned at the back of the anesthetized rats head, with the Z-axis of the sensor parallel to the rat's head. Through the sensor's Z-axis direction, weak magnetic fields emanating from the rat's head can be measured. Plastic tubes are fixed near the ears and connected to an external speaker, allowing sound stimuli to be transmitted into the rat's ear canal through the tubes. (d) The homemade SERF magnetometer. The shell is made of high-temperature resistant and non-magnetic nylon material. The center of the vapor cell is 4 mm away from the shell. The figure has been authorized by Hangzhou Q-Mag Technology Co., Ltd. (e) Passive single-tone stimulation paradigm with red regions in the stimulated state, blue regions in the silent state, and ISI as the distance between the two regions. (For interpretation of the references to color in this figure legend, the reader is referred to the Web version of this article.)

As shown in Fig. 1 (a), the internal structure of the SERF AM, primarily composed of an optical path, a vapor cell and a detector. The light first emanates from the laser, passes through the vapor cell, and is received by a photodiode (PD). This signal is then processed into an electrical signal, ultimately received by a personal computer (PC). The laser light is shaped and expanded to enhance the interaction between more atoms and photons, thereby increasing the magnetometer's signal amplitude. In this case, we are using an elliptically polarized light magnetometer. Compared to the circularly polarized light magnetometer, which typically employs a single PD for light absorption detection and is prone to significant noise influenced by the intensity of incident light, the elliptically polarized light magnetometer uses a balanced detector for detecting the emerging elliptically polarized light, greatly reducing light intensity noise and common-mode noise in the system. This balanced detector consists of a half-wave plate, a wollaston prism and two PDs. The wollaston prism splits the incident light into vertical and horizontal polarization components, which are detected by the PDs. Changes in the difference between these two polarization components are detected through differential means. To achieve elliptically polarized light, a combination of a linear polarizer (LP) and a quarter-wave plate is used, where the elliptically polarized light is represented as a combination of left and right circularly polarized light (Refer to Supplementary Fig S2 for a detailed schematic illustrating the rotation of elliptically polarized light in the polarized atomic vapor cell).

Finally, after a series of optimizations to the signal from the differential photodetector, a bandwidth ranging from 1 to 80 Hz and a sensitivity of 20 fT/ $\sqrt{\text{Hz}}$ for the SERF magnetometer is achieved. For further details, please refer to the Supplementary Information (Supplementary Text, Fig S3-S4).

Neural activities in the brain produce faint magnetic fields. When neurons are active, they generate currents through electrochemical processes. The magnetic fields produced by these current flows can penetrate the brain and skull, detected by highly sensitive magnetic field sensors placed near the scalp. Despite these magnetic fields being extremely weak, the use of atomic magnetometers, which are highly sensitive magnetic field detection devices, allows for the precise measurement of these fields. Like other small rodent animals, rats have the characteristic of lacking brain sulci; their cerebral cortex surface is smooth without convolutions, causing cortical currents tend to flow normal (radial) to the skull. According to previous studies, for a uniform spherical conductor, radial current dipoles do not produce a measurable magnetic field outside of the sphere due to the balance between the main current and the return current. However, the shape of a rat's brain significantly differs from a sphere, and the main currents may not be entirely radial, hence a measurable magnetic field could exist outside the head [33,34]. We positioned the MEG sensors on the dorsal surface of the skull, in a plane parallel to the expected direction of current flow in the auditory cortex, and were able to detect magnetic fields produced by the main or return currents induced by auditory stimuli. Notably, using this non-invasive approach, we successfully obtained clear auditory evoked fields without resorting to more invasive methods. For determining depth of anesthesia, we monitor the condition of animals by assessing the heart rate, respiratory rate, and physical behaviors of rats [35] (Supplementary Text, Fig S5-S6).

## 3 Experimental methodology

### 3.1 Animals

The procedures for the breeding and use of rats were approved by the Ethics Committee of Zhejiang University of Technology (Approval No. 20230703017), and all experimental procedures followed the principles and guidelines for the care and use of animals established by the government and relevant organisations. Balb-c rats, weighing about 180 g, were purchased from Zhejiang Chinese Medical University Laboratory Animal Research Center. The rats were fed freely for more than one week. On the day of the experiment, the rats preoperative fasting 12 h, 12 h water. Before the experiment, rats were anesthetized with Pelltobarbitalum Natricum at a dose of 40 mg/kg, and the experiment was performed 10 min after anesthesia.

### 3.2 Experimental procedure

The original design of the experiment is shown in Fig. 1 (b). After anesthesia, rats were placed inside a magnetic shielding barrel, and their brain magnetism was measured using a specially constructed experimental platform. Fig. 1 (c) shows the actual equipment used for measurements and the position between the sensor and the rats brain during measurement. After anesthetizing a rat weighing 180 g, to more intuitively determine the distance between the sensor and the scalp and to reduce the temperature around the air chamber causing damage to the rat's fur, the hair on the rat's head was shaved off. Subsequently, the rat's limbs were secured to a foam board using medical tape. This procedure aims to minimize the impact of occasional spontaneous trembling that may occur after anesthesia. Fig. 1 (d) features the SERF magnetometer used in the measurements, where the large optical path is integrated and reduced, resulting in a sensor width of only 25 mm and a distance of only 4 mm from the vapor cell to the casing. The miniaturized magnetometer facilitates multi-array measurements and plays an important role in brain source localization. It also supports wearable implementation, allowing subsequent measurements to be closer to the cerebral cortex and capture weaker magnetic fields. Subsequently, we secured the SERF AM device onto a specially designed experimental setup, positioning the sensor's Z-axis direction on the dorsal side of the rat's skull top, approximately 1 mm from the rats scalp. The plane of the sensor was thus oriented parallel to cortical columns within the auditory cortex, which lies along the lateral surface of the brain in rat. Due to the constraints of the shielding cylinder, the auditory stimuli were delivered to the animal's ear through a 5 cm long tube with an internal diameter of 1.5 mm. The custom long tube was connected to a speaker and calibrated for sound. The tube was inserted into the rat's ear canal and sealed with tape to ensure the sound entered the rat's ear as much as possible. For sound frequency modifications, we created audio files for different frequencies within the rats hearing range (2.3 kHz, 4.3 kHz, 6.3 kHz, 8.3 kHz) and stored them in the speaker. The sound output was controlled by the pulse signal of the signal source (the ISI size was controlled by adjusting the duty cycle of the signal source, at 50 %, 75 %, 83.3 %, 90 %). The loudness of each frequency was maintained at 90 dB, with a stimulus duration of 1 s. The stimulation method, as shown in Fig. 1 (e), involves the pulse signal being high (5 V) for a silent state and low (0 V) to trigger stimulation, with the overall task comprising a stimulus sequence formed by different ISI and frequencies.

In the experiment concerning the auditory evoked fields in rats, a total of six rats were used. Two of these rats were utilized in preliminary investigations to ascertain whether magnetic field signals could be measured. The remaining four rats corresponded to four different stimulus intervals and four different sound frequencies. The objective was to prevent the variance between different stimulus intervals from affecting the cognition and experimental conditions of the same rat. For each stimulus interval, three sets of data measurements were conducted on a single rat (with the intervals between consecutive stimuli tests spanning several days to prevent neural fatigue from frequent testing, which could affect the data). The three sets of data measured from the same rat were then superimposed and averaged to reflect consistency and reliability.

### 3.3 Data processing

The signals from the sensor are collected through a National Instruments acquisition card (USB-6281). The sampled signals are digitally bandpass filtered from 0.1 to 40 Hz, after which the data are segmented according to the intervals of the stimuli. The segmented data are divided into 100 groups, each corresponding to the magnetic field signals evoked by each stimulus. By summing and averaging the signals from these 100 groups and applying smoothing techniques, the ERMFs are analyzed. The amplitude of each data point is measured starting from the baseline, which is chosen as the average amplitude during the 200 ms period before the presentation of the sound. The purpose of the data analysis is to extract the weaker magnetic field signals induced by the stimuli from a combination of environmental and physiological noise. The smoothing step has minimal impact on the final MEG signals but is necessary to mitigate the effects of occasional spike signals caused by environmental noise. To achieve the highest signal-to-noise ratio in the processed data, three sets of data are recorded under each stimulus interval. Averaging these three sets of MEG data measured at different times yields a signal with an improved signal-to-noise ratio. Finally, we employed a one-way Analysis of Variance (ANOVA) to analyze the amplitudes under the four ISI and sound frequencies. In this experiment, ISI and frequencies were considered as inter-subject factors to evaluate the amplitude of the rats' ERMFs, ensuring comparability of the results.

## 4 Results

According to Table 1, amplitude variations were observed under different frequencies and ISI. Subsequent analyses were based on data recorded from four rats. The experiment was divided into two parts, analyzing the impact of ISI and frequency on amplitude under the passive single-tone paradigm. Four major groups were established for different sound frequencies (2.3 kHz, 4.3 kHz, 6.3 kHz, 8.3 kHz), with each frequency having four corresponding ISI (3 s, 5 s, 7 s, 9 s).

**Table 1:** Amplitudes of M300 at different frequencies in the passive pure tone paradigm. The table includes data for four frequencies, each with four stimulus intervals. These intervals correspond to the data from four rats, with each interval containing three sets of measurement data from a single rat.

Frequency	Inter-Stimulus Interval/s			
	3	5	7	9
2.3 kHz	Amplitude/pT	Amplitude/pT	Amplitude/pT	Amplitude/pT
	−0.0071	−0.0092	−0.0606	−0.0755
	−0.0038	−0.0149	−0.0549	−0.0789
	−0.0048	−0.0173	−0.0494	−0.0887
4.3 kHz	Amplitude/pT	Amplitude/pT	Amplitude/pT	Amplitude/pT
	−0.0289	−0.0430	−0.1023	−0.1050-
	−0.0160	−0.0427	−0.0818	−0.0608
	−0.0269	−0.0588	−0.0867	−0.1564
6.3 kHz	Amplitude/pT	Amplitude/pT	Amplitude/pT	Amplitude/pT
	−0.1908	−0.1451	−0.2112	−0.1333
	−0.1147	−0.1288	−0.1782	−0.1455
	−0.1179	−0.1733	−0.1977	−0.1454
8.3 kHz	Amplitude/pT	Amplitude/pT	Amplitude/pT	Amplitude/pT
	−0.1059	−0.2225	−0.2432	−0.1690
	−0.1644	−0.1888	−0.2074	−0.2224
	−0.1155	−0.1714	−0.1971	−0.2381

**Fig. 2:**
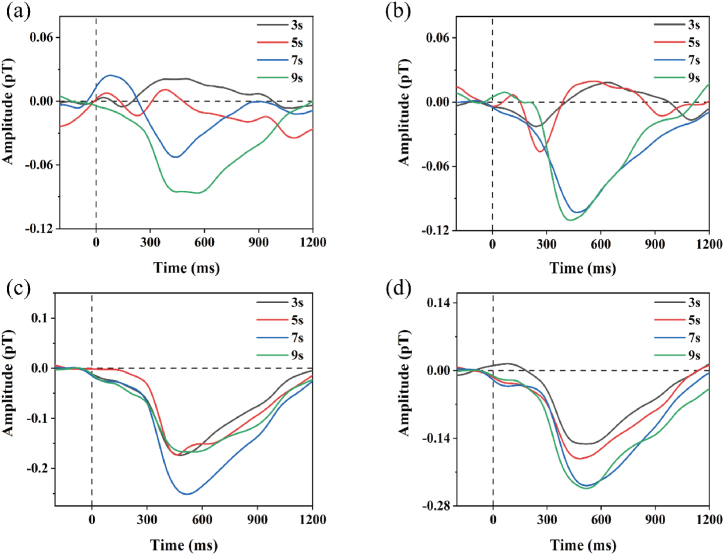
(a)–(d) represent the aggregated average ERMFs for four rats under four auditory frequencies (2.3 kHz, 4.3 kHz, 6.3 kHz, 8.3 kHz). The X-axis indicates the latency in ms, while the Y-axis shows the amplitude in pT. Four different colored lines represent different ISI (3 s, 5 s, 7 s, 9 s). The amplitude becomes larger with changes in ISI and frequency.

Stimuli, each lasting 1 s, were presented for every ISI and frequency, and average ERPs were recorded, as shown in Supplementary Fig S8. The information depicted in the figure illustrates that under passive single-tone stimulation, each frequency and ISI could evoke corresponding negative waveforms, displaying relatively stable stimulus cycles with adequate signal-to-noise ratios. Each amplitude of the ERMFs was quantified as the maximum extremum within a small time window around the latency period (window boundaries: M300 from 300 to 500 ms). We utilized a nomenclature similar to that used in human studies, labeling the approximate latency period as M300. In Fig. 2 (a) - (d), the amplitude of the averaged ERMFs from three samples revealed that, regardless of the frequency, the M300 amplitude increased with the regular periodic presentation of each ISI.

**Fig. 3:**
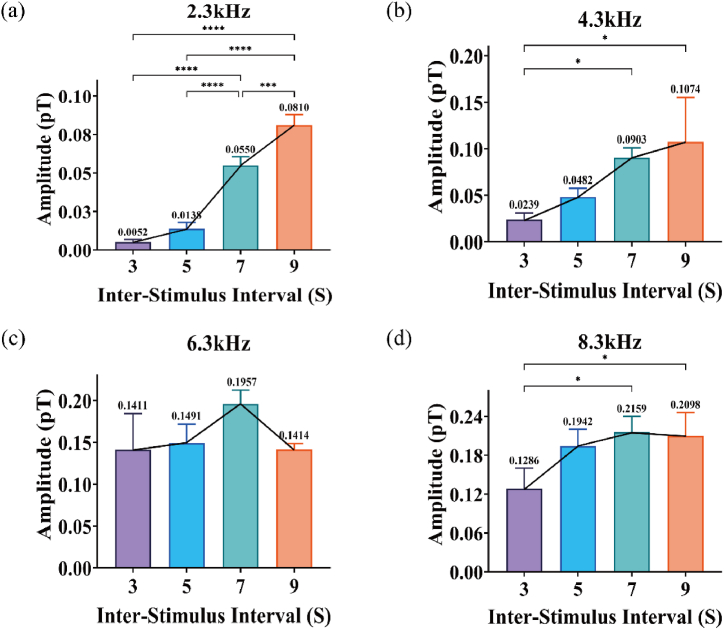
(a)–(d) display the average values ± SEM of ERMFs for four rats at each ISI under four sound frequencies (2.3 kHz, 4.3 kHz, 6.3 kHz, 8.3 kHz), with different colors corresponding to different ISI (3 s, 5 s, 7 s, 9 s). The amplitude of M300 increases with the increase in ISI. (For interpretation of the references to color in this figure legend, the reader is referred to the Web version of this article.)

For a more intuitive analysis, we extracted extrema within the specified time window and presented them in the form of histograms. Overall, an increasing trend was observed. The amplitude of the M300 component was significantly greater at longer ISI compared to shorter ISI. As shown in Fig. 3 (a) - (d), through statistical analysis, we elucidated the impact of ISI on M300 component amplitudes. At lower frequencies, amplitudes increased rapidly within shorter ISI ranges, while the amplitude increase within longer ISI was relatively moderate, showing a trend toward saturation. When the ISI exceeded 5 s, the amplitude change became more pronounced. Specifically, ANOVA analysis of the histogram data revealed significant effects. At 2.3 kHz (F (3, 8) = 154.2, P < 0.0001), 4.3 kHz (F (3, 8) = 6.910, P = 0.0130), and 8.3 kHz (F (3, 8) = 5.420, P = 0.0250) frequencies, significant differences were observed in M300 amplitudes. However, at 6.3 kHz, the ISI did not show a significant difference in M300 amplitude.

**Fig. 4:**
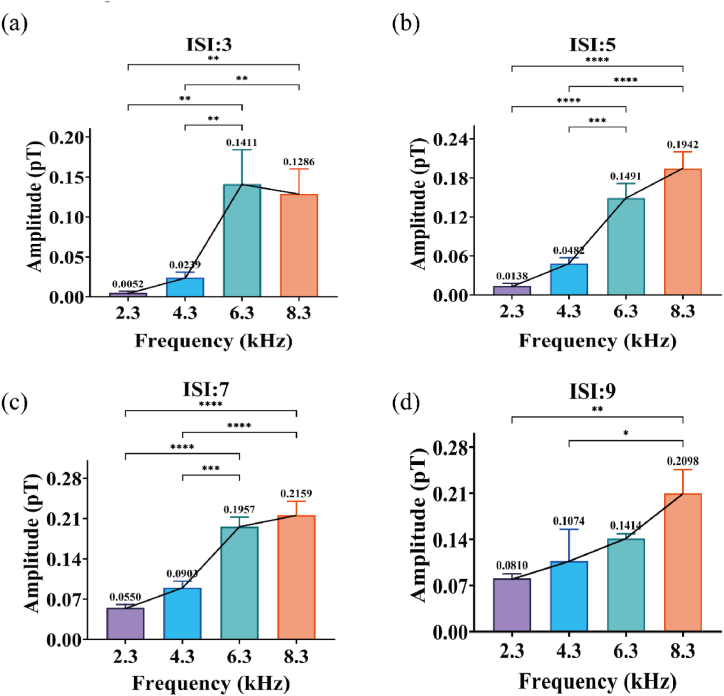
(a)–(d) represent the aggregated average ERMFs for four rats under four auditory frequencies (2.3 kHz, 4.3 kHz, 6.3 kHz, 8.3 kHz). The X-axis indicates the latency in ms, while the Y-axis shows the amplitude in pT. Four different colored lines represent different ISI (3 s, 5 s, 7 s, 9 s). The amplitude becomes larger with changes in ISI and frequency.

Finally, we conducted a similar analysis to examine the influence of frequency on the M300 component. As shown in Fig. 4 (a) - (d), it was observed that, under the same ISI, varying frequencies led to an increasing trend in the amplitude of the M300 component in rats. Particularly, at higher frequencies, the impact of frequency on amplitude was more significant within the same range of ISI. Specifically, at an ISI of 3 s (F (3, 8) = 20.39, P = 0.0004), 5 s (F (3, 8) = 66.66, P < 0.0001), 7 s (F (3, 8) = 73.76, P < 0.0001), and 9 s (F (3, 8) = 10.08, P = 0.0043), significant differences in amplitude were observed as the stimulus frequency increased under the passive single-tone paradigm.

The existing results reveal that passive monaural stimulation of anesthetized rats can generate the task-related component M300, without the need for active task participation. In this study, we observed that with changes in the ISI, the variations in amplitude are quite pronounced, especially when the ISI shifts from 3 s to 5 s. We infer that rats possess a recovery function similar to humans, with a recovery period of 5 s, which is shorter compared to the human recovery period (6–10 s) [36,37]. As the ISI increases, the increase in amplitude reflects fewer refractory states in the brain regions [[38], [39], [40]]. Previous EEG reports have mentioned corresponding recovery functions, where the refractory state and the recovery function itself reflect certain aspects of auditory sensory memory [[41], [42], [43]]. In the study of frequency effects, the M300 component, indicative of sound detection and associated with selective attention, is related to attention-grabbing features. When attention is actively directed towards behaviorally relevant auditory stimuli, the incoming stimulus continually updates the brain's internal representation of the acoustic environment based on specific attention or targets.

## 5 Conclusions and discussion

In summary, we employed OPMs with a sensitivity of 20 fT/ $\sqrt{\text{Hz}}$ as a neurophysiological research tool to ERMFs in rats under a passive stimulation paradigm, exploring amplitude variations from the perspectives of ISI and frequency. The results indicated noticeable waveform amplitude changes, especially between 3 and 5 s, suggesting a shorter auditory recovery period in rats compared to humans. Moreover, the M300 component, associated with selective attention, increased with frequency, indicating rats have higher sensitivity to high-frequency sounds, shedding light on their auditory system's sensitivity and processing mechanisms for different frequencies. These findings provide significant insights into understanding rat auditory processing mechanisms, especially in terms of recovery function and attention. The emergence of the M300 component may reflect memory updating operations, with the amplitude of M300 significantly impacted when stimuli are presented with relatively long ISI. At shorter ISI, due to divided attention and insufficient recovery time, neurons have a shorter period to return to a state ready to respond to a subsequent stimulus. Under these shorter ISI, neurons may still be in a state of incomplete recovery, leading to diminished responses. This neural response is doubly influenced, manifesting as a trend towards attenuation. However, with a stimulus interval of 5 s, appropriate attentional adjustment and recovery time allow neurons more time to fully recover, thereby making the neural response to each stimulus more pronounced. We propose that the magnitude of M300 reflects the attention allocation process, with the temporal interval and frequency of auditory stimuli appearing to be primary determinants of M300 amplitude. This is because they directly affect the allocation of attentional resources during task execution. In passive single-stimulus paradigms, the time between target stimuli is a critical factor as it determines how efficiently the rats nervous system processes key information. Finally, we propose that using OPMs combined with a passive stimulation paradigm to explore sensory processing and attention mechanisms in rats offers a new approach for subsequent human neurophysiological research, especially for treatment in less convenient populations. Due to the limited number of channels used in this study and the restrictions imposed by the sensor size and rat head, only a single-channel sensor was utilized. Future research should increase the number of sensor channels for multi-point measurements, expand to multi-dimensional measurements, and replicate the same stimulation paradigm in human models for a more detailed comparison and better understanding of the differences between these two species.

## Funding

This work was supported in part by 10.13039/501100001809National Natural Science Foundation of China (U20A20219, 61805213); 10.13039/501100004731Natural Science Foundation of Zhejiang Province (LQ23H160032).

## Data availability statement

Data underlying the results presented in this paper are not publicly available at this time but maybe obtained from the authors upon reasonable request.

## CRediT authorship contribution statement

**Yi Ruan:** Writing – review & editing, Supervision, Funding acquisition. **Zhao Xiang:** Writing – original draft. **Guanzhong Lu:** Investigation. **Yuhai Chen:** Methodology. **Yufei Liu:** Project administration. **Fan Liu:** Resources. **Jiahao Wang:** Software. **Ying Zhang:** Visualization. **Jia Yao:** Conceptualization. **Yu Liu:** Data curation. **Qiang Lin:** Validation, Funding acquisition.

## Declaration of competing interest

The authors declare that they have no known competing financial interests or personal relationships that could have appeared to influence the work reported in this paper.
