# Supplementary material for: Non-invasive measurement of rat auditory evoked fields using an optically pumped atomic magnetometer: Effects of task manipulation
Source: Heliyon. 2024 May 23;10(11):e31740. doi: 10.1016/j.heliyon.2024.e31740 (PMC11152962; doi:10.1016/j.heliyon.2024.e31740)
Supplement: Multimedia component 1 [file mmc1.docx]

Non-invasive Measurement of Rat Auditory Evoked Fields Using an Optical Atomic Magnetometer: Effects of Task Manipulation of primary manuscript: supplemental document

**This PDF file includes:**

Supplementary information Text

Figs. S1 to S7

**SERF AM:**

The external magnetic field was realized by a pair of Helmholtz coils with a diameter of 5 cm and 150 turns, and its inductance is about 20 mH. The actual measurement shows that the 240 mA current in the coil center region corresponds to a 1.25 mT magnetic field, which was basically consistent with the simulation calculation of COMSOL Multiphysics. Fig. S1 shows the magnetic field distribution in the central plane of parallel and vertical coils respectively. Fig. S2 provides a visual representation of the process when elliptically polarized light passes through the polarized atomic gas cell. Before measurements, sensitivity and bandwidth assessments were conducted on the single-light spin-exchange relaxation-free (SERF) atomic magnetometer, as depicted in Figures Fig. S3 and Fig. S4. The sensitivity reached 20 fT/Hz, and the measurement response bandwidth, at -3 dB, was approximately 80 Hz. Fig. S5-S7 include the data table of heart rate and respiratory rate for rats after anesthesia, error analysis graphs for respiratory and heart rates, and magnetocardiograms under anesthesia.


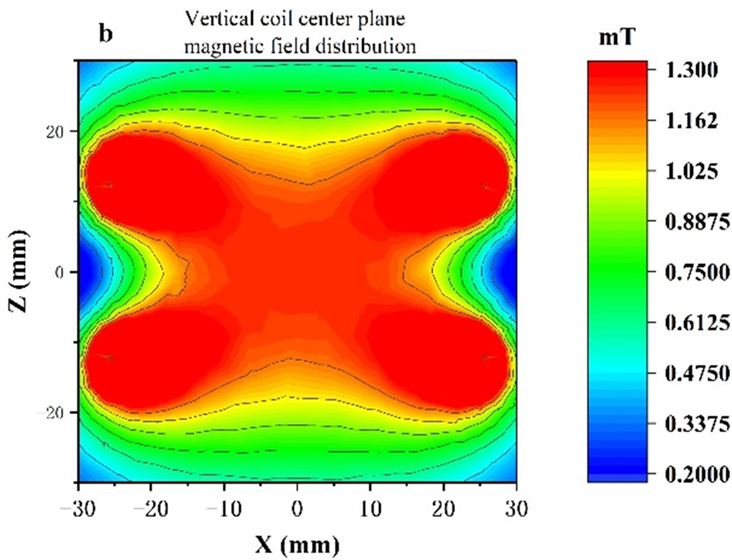

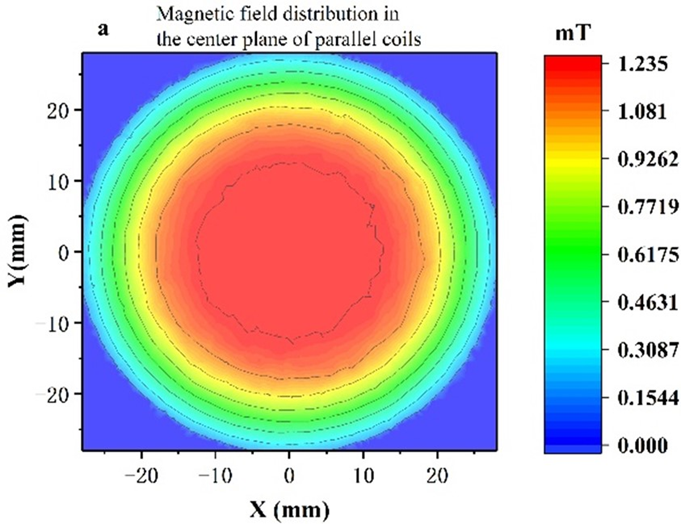


**FIG. S1 Magnetic field distribution of the external magnetic field coils .**


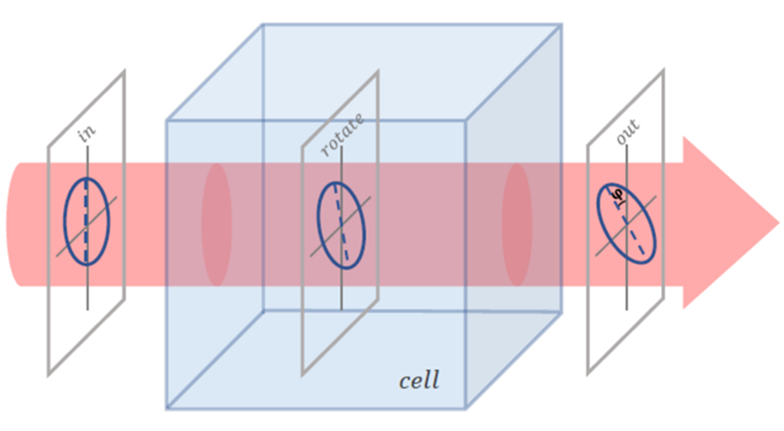


**
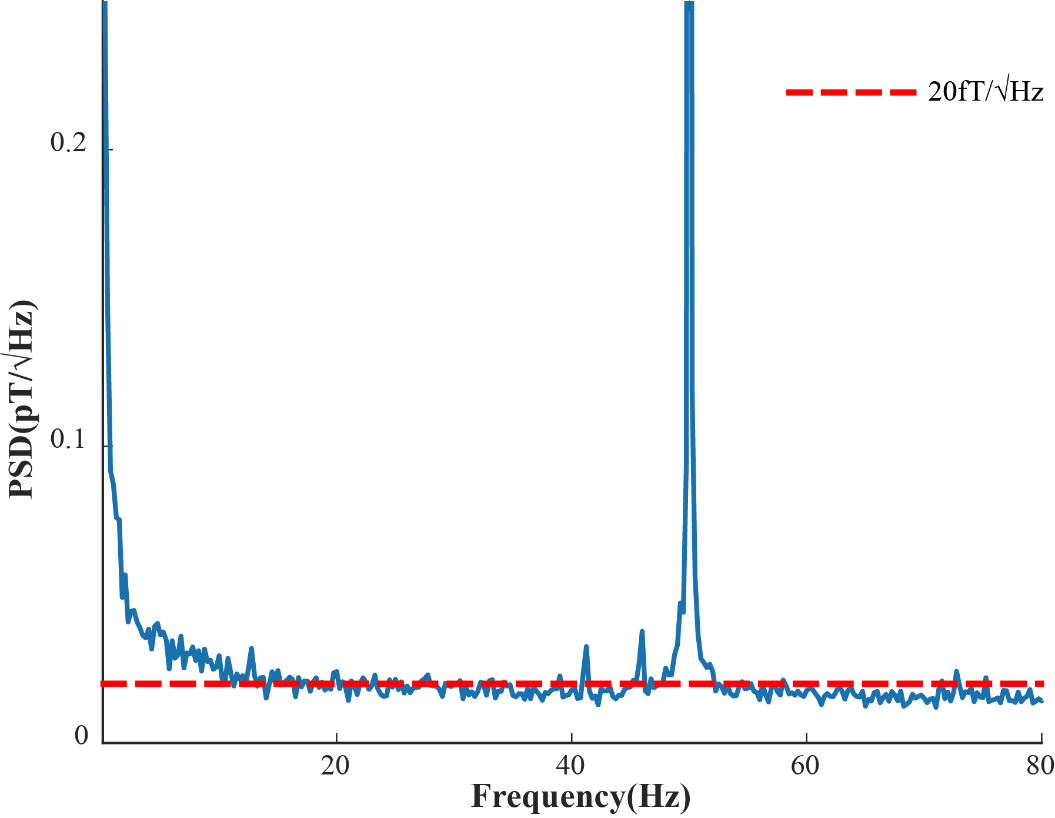
FIG. S2 Schematic of elliptically polarized light rotating in a polarized gas cell.**

**FIG. S3 Frequency response curve with the amplitude beginning at 500 pT.**

**
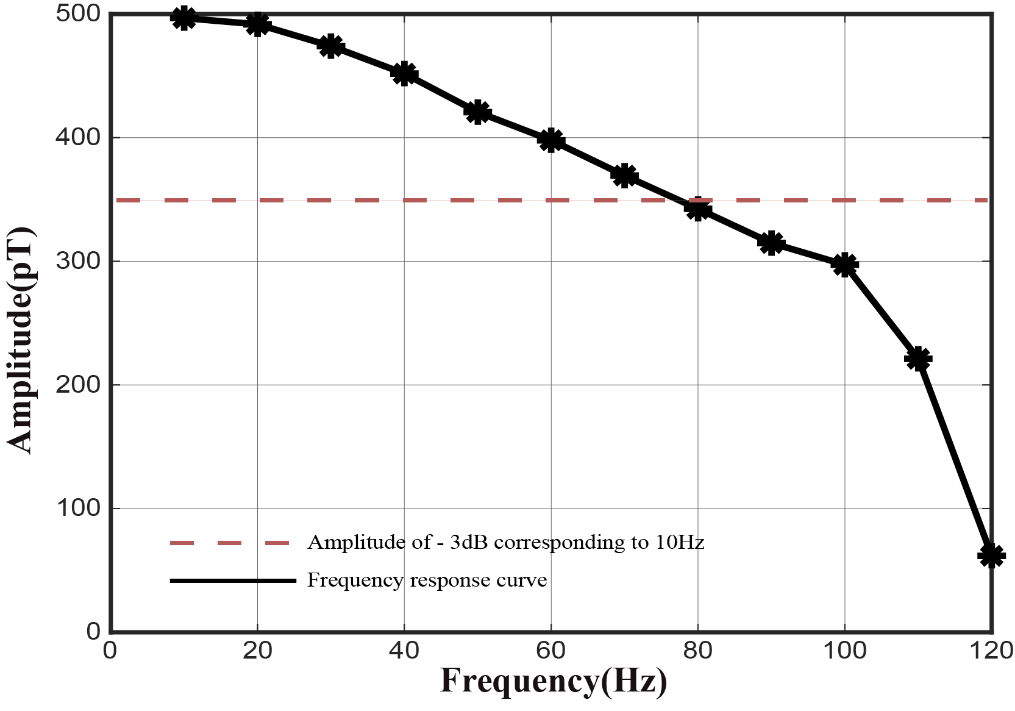
**

**FIG. S4 Sensitivity of SERF atomic magnetometer.**


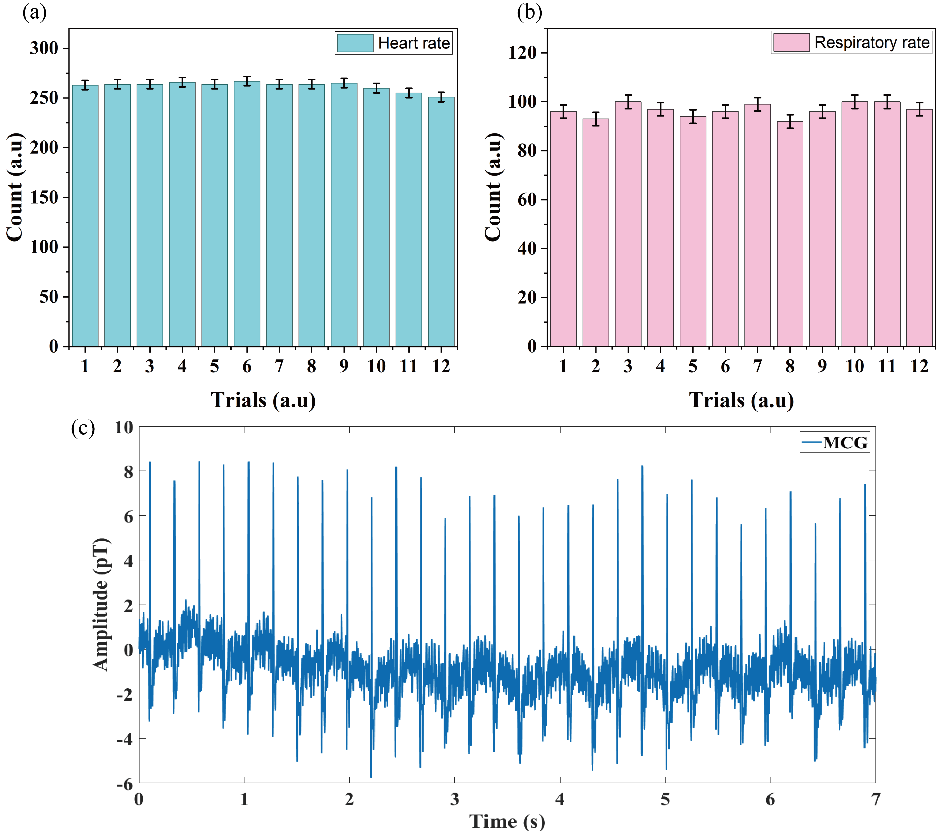


**FIG. S5 Heart rate and respiratory monitoring graphs for anesthetized rats. (a) The heart rate of the anesthetized rat tends to stabilize, maintaining around 260 beats per minute, (b) The respiratory rate of the anesthetized rat remains within the normal range of 90-100 breaths per minute, (c) The rat's magnetocardiogram shows a stable overall cardiac magnetic signal.**


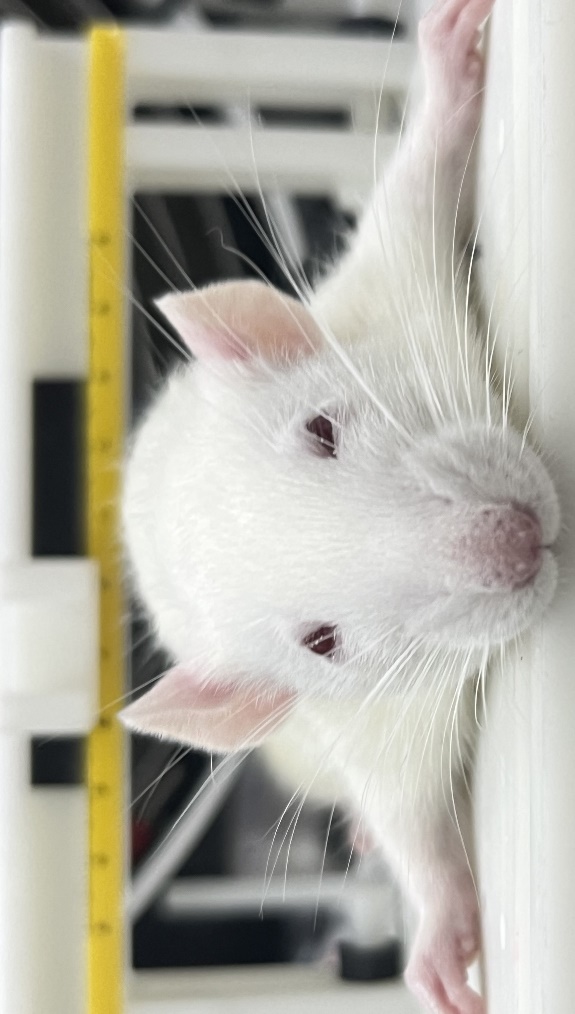


**FIG. S6 Overall condition of the anesthetized rat: the eyes are in a normal state, and the color of the paws and ears is normal pink.**

**Table. S7 Data on heart rate and respiratory rate of rats under anesthesia**

| Heart rate | | Respiratory rate | |
| --- | --- | --- | --- |
| 263 | 255 | 96 | 97 |
| 264 | 260 | 93 | 100 |
| 266 | 265 | 100 | 100 |
| 264 | 264 | 97 | 96 |
| 267 | 264 | 94 | 92 |
| 264 | 251 | 96 | 99 |

**Results:**


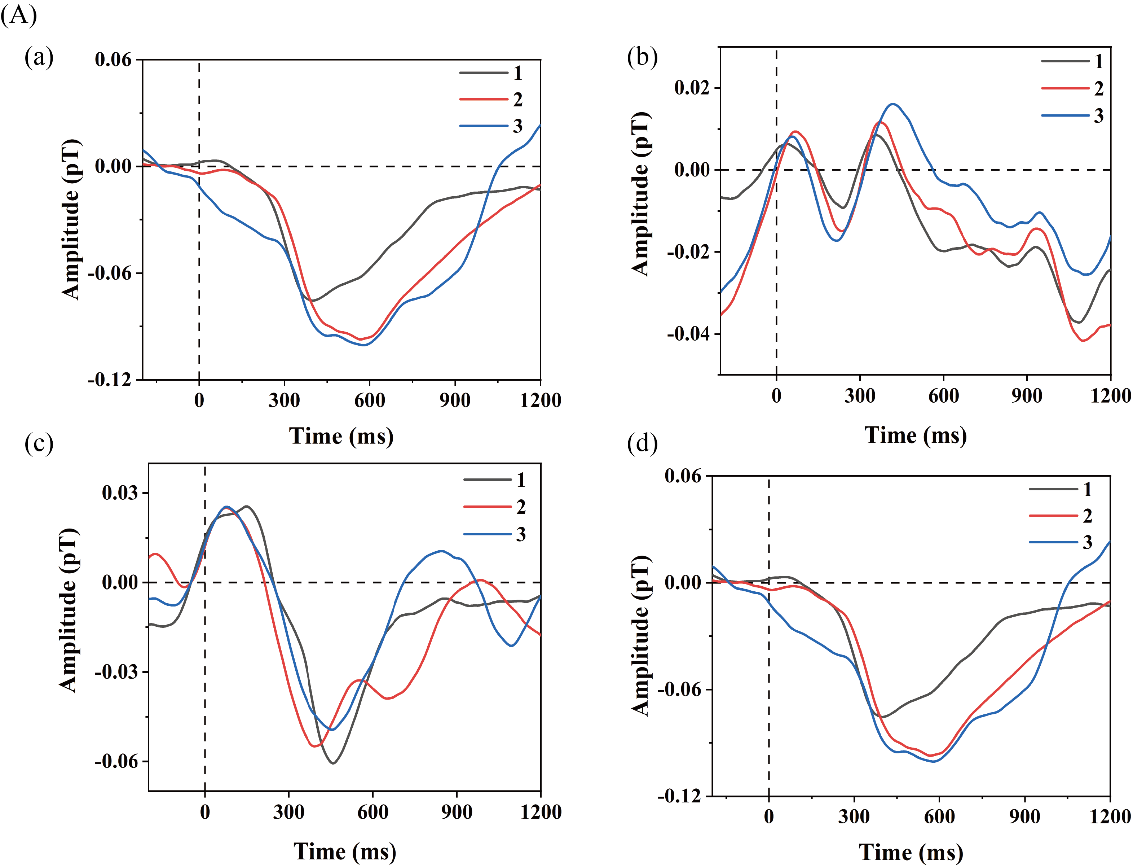
 Fig. S8 presents waveforms corresponding to different frequencies and stimulus intervals.


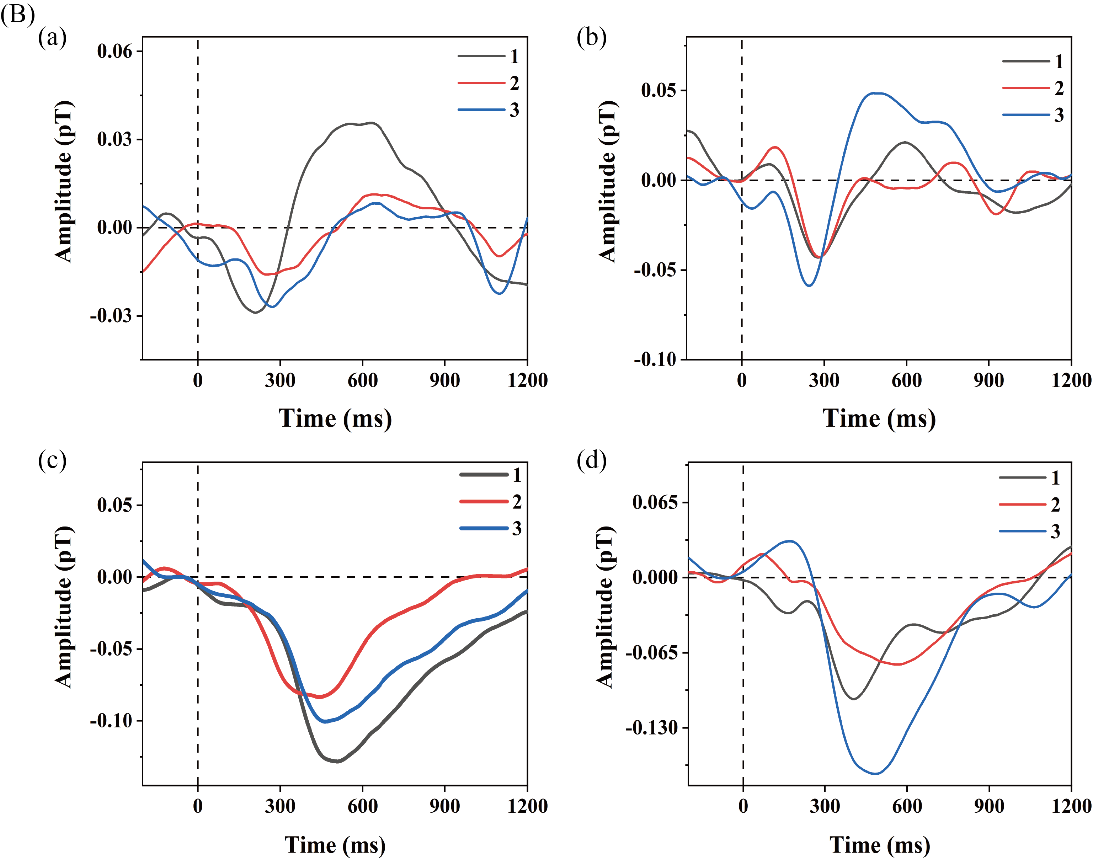


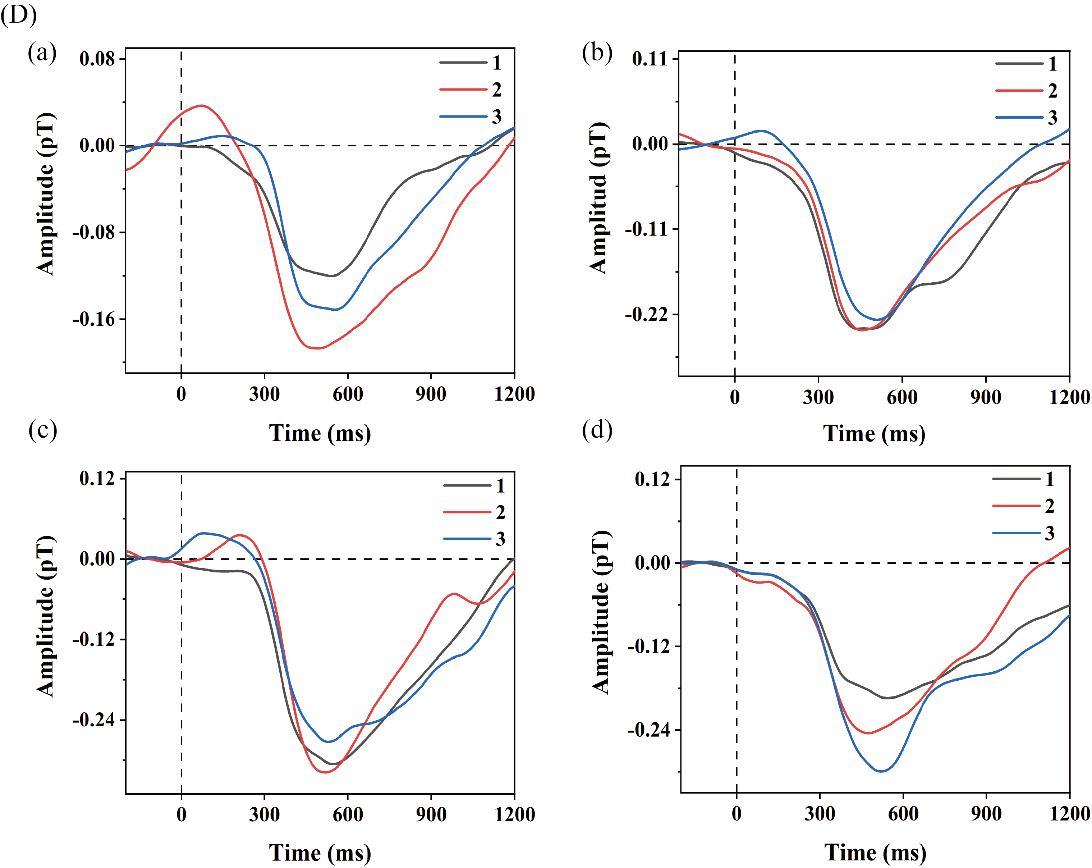

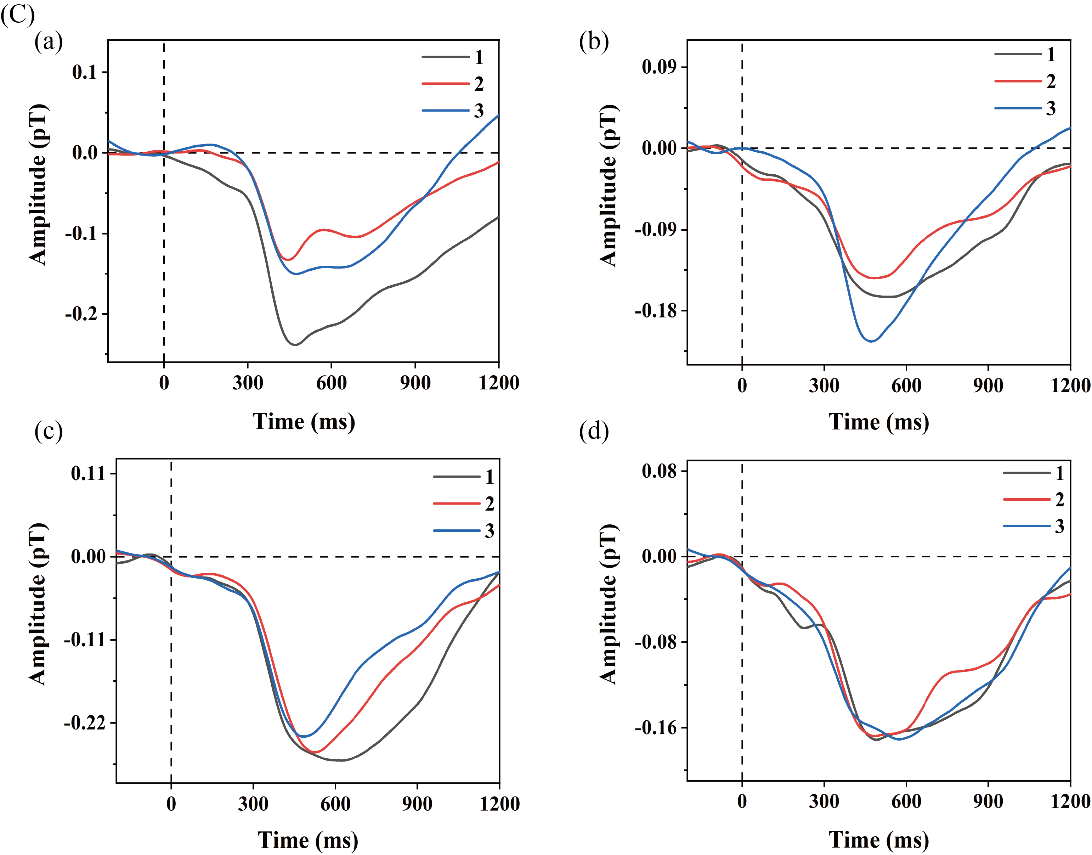


**FIG. S8 shows waveforms corresponding to different frequencies and stimulus intervals. A, B, C, and D represent waveforms at four frequencies (2.3 kHz, 4.3 kHz, 6.3 kHz, 8.3 kHz) and various stimulus intervals (3 s, 5 s, 7 s, 9 s), respectively. The three curves represent the event-related magnetic field waveforms induced in each rat under the passive single-stimulus paradigm at the same stimulus intervals.Under each stimulus interval, three sets of measurement data from a single rat are included.**
